# Postoperative dexamethasone administration following posterior spinal fusion for adolescent idiopathic scoliosis: A systematic review of worldwide data

**DOI:** 10.1016/j.xnsj.2026.100891

**Published:** 2026-04-18

**Authors:** Fahad H. Abduljabbar, Rakan Bokhari, Raed M. Sharaf, Abdulrheem A. Almokhtar

**Affiliations:** a Department of Orthopaedic Surgery, Faculty of Medicine, King Abdulaziz University, Jeddah, Saudi Arabia; b Department of Surgery, Faculty of Medicine, King Abdulaziz University, Jeddah, Saudi Arabia; c Faculty of Medicine, King Abdulaziz University, Jeddah, Saudi Arabia; d Orthopaedic Surgery, King Abdullah Medical City, Makkah, Saudi Arabia

**Keywords:** Opioid consumption, Postoperative pain, Nausea, Wound infection, Dexamethasone, Adolescent idiopathic scoliosis, Surgical site infection

## Abstract

**Background:**

Dexamethasone reduces postoperative pain and opioid consumption in various surgical populations; however, its role in adolescents undergoing posterior spinal fusion (PSF) for adolescent idiopathic scoliosis (AIS) remains unclear**.**

**Methods:**

A systematic search of PubMed, Web of Science, Scopus, and Google Scholar was conducted for studies published between 2015 and 2024. Comparative studies evaluating perioperative or postoperative dexamethasone use in AIS patients undergoing PSF were included. Non-AIS populations and noncomparative studies were excluded. Study quality was assessed using the Revised Cochrane Risk of Bias Tool and the Newcastle–Ottawa Scale.

**Results:**

Four studies involving 614 patients were included. Dexamethasone was associated with reduced postoperative pain, opioid consumption, hospital length of stay, and postoperative nausea and vomiting. No statistically significant difference in surgical site infection (SSI) was observed between dexamethasone-treated and control groups. However, heterogeneity across studies limited quantitative pooling, and available data were likely underpowered to detect small differences in SSI risk.

**Conclusions:**

Postoperative dexamethasone administration following posterior spinal fusion for adolescent idiopathic scoliosis appears to improve postoperative recovery parameters, including pain control and opioid reduction. However, current evidence remains inconclusive on surgical site infection risk, and available studies are likely underpowered to detect rare complications.

## Highlights

• This systematic review evaluated dexamethasone use in adolescents undergoing posterior spinal fusion for AIS.
• Dexamethasone was associated with reduced postoperative opioid consumption, pain scores, and hospital length of stay.
• No statistically significant increase in surgical site infection was identified.
• Variability in dosing protocols highlights the need for standardized, adequately powered prospective trials.

## Introduction

Adolescent idiopathic scoliosis (AIS) is a 3-dimensional spinal deformity of unknown etiology that typically presents between 10 and 18 years of age. The reported prevalence ranges from 0.47% to 5.25%, with a higher incidence in females and female-to-male ratios increasing in patients whose curves progress to surgical intervention [[1], [2], [3],^28^]. Management strategies are guided by curve magnitude and skeletal maturity. Mild curves (10°–25°) are generally monitored, whereas moderate curves (25°–40°/45°) may be treated with bracing. Surgical intervention, most commonly posterior spinal fusion (PSF), is indicated for progressive curves exceeding 40°–45° in skeletally immature patients [3].

Although PSF effectively corrects spinal deformity and stabilizes the spine, it is associated with substantial postoperative pain, inflammatory response, and perioperative morbidity. Consequently, optimization of perioperative management has become a central focus in AIS surgery to enhance recovery and reduce complications [4].

Dexamethasone, a potent synthetic glucocorticoid, possesses anti-inflammatory, antiemetic, and analgesic properties [5]. In diverse surgical populations, perioperative dexamethasone has been shown to reduce postoperative pain, opioid consumption, and postoperative nausea and vomiting (PONV). A meta-analysis of 60 randomized controlled trials involving 6,696 patients demonstrated that 4–5 mg of dexamethasone significantly reduced 24-hour PONV rates compared with placebo [6]. Additional studies across abdominal, orthopedic, and high-risk surgical procedures have similarly reported reductions in pain scores and inflammatory markers following dexamethasone administration [[9], [10], [11]].

The pharmacologic effects of dexamethasone are mediated through inhibition of the nuclear factor-kappa B pathway, suppression of proinflammatory cytokines, and stabilization of cellular membranes [7]. While these mechanisms suggest potential benefit in AIS patients undergoing PSF, glucocorticoid-induced immunosuppression raises theoretical concerns regarding impaired wound healing and increased surgical site infection (SSI) risk [12]. Importantly, prior analyses in nonspine surgical populations have not demonstrated a consistent increase in postoperative infection rates [13].

Despite growing interest in multimodal analgesia protocols for AIS surgery, evidence specifically evaluating dexamethasone use in adolescents undergoing PSF remains limited and heterogeneous. Therefore, the purpose of this systematic review was to evaluate the safety and efficacy of dexamethasone administration in patients with AIS undergoing posterior spinal fusion, with particular attention to infection risk and postoperative recovery outcomes.

## Methods

### Study design

This systematic review was carried out in accordance with the “Preferred Reporting Items for Systematic Reviews and Meta-Analyses” guidelines [14].

### Search strategy

A comprehensive literature search was performed using PubMed, Embase, Cochrane Library, Web of Science, and Scopus to identify relevant studies published between January 2015 and December 2024. Google Scholar and reference lists of included articles were additionally screened to ensure comprehensive capture of eligible studies. A combination of various keywords and their synonyms was used for searching the databases. The keywords used for database search included “*Dexamethasone,”* “*Decadron,”* “*Scoliosis,”* “*Posterior Spinal Fusion,”* These keywords were combined using the “AND” and “OR” operators. Supplemental File I provides complete details on the keywords used in the database search for this systematic review.

### Study eligibility

Eligibility criteria were defined using the PICOS framework. The population included adolescents with idiopathic scoliosis undergoing posterior spinal fusion. The intervention was perioperative or postoperative dexamethasone administration, compared with placebo or no dexamethasone. Outcomes included surgical site infection, length of hospital stay, opioid consumption, postoperative pain scores, and postoperative nausea and vomiting. Eligible study designs included randomized controlled trials and prospective or retrospective comparative studies.

### Inclusion criteria

Studies were included if they involved patients with adolescent idiopathic scoliosis undergoing posterior spinal fusion, evaluated perioperative or postoperative dexamethasone administration, included a comparator group, and reported at least 1 prespecified outcome of interest.

### Exclusion criteria

Studies were excluded if they involved adult degenerative scoliosis, neuromuscular scoliosis (eg, cerebral palsy), or other non-AIS populations; included noncomparative designs such as case reports or case series; involved spinal procedures other than posterior spinal fusion; or were published in languages other than English.

### Study selection

After retrieving the results from the database search, the final files were exported to Rayyan, a software specifically designed for the screening process of systematic reviews [15]. Duplicates were detected and removed before the screening process. Two independent reviewers who were blinded to each other’s decisions were involved in the screening process. The initial screening was performed based only on titles and abstracts. After study selection, the 2 reviewers compared their decisions, and in case of any conflicts, agreed on a decision based on mutual discussion. Should any disagreement persist, a third reviewer was involved in finalizing the decision. Full-text article screening was performed. After the final study selection, data regarding patient demographics and study outcomes were extracted using an Excel spreadsheet.

### Quality assessment

Two independent reviewers assessed the risk of bias. The Newcastle–Ottawa Scale (NOS) was used to assess the quality of retrospective and prospective studies. The NOS checklist has 8 questions divided into 3 categories. These categories included participant selection, comparability focusing on controlling risk factors, and outcomes, which included follow-up duration and outcome assessment. The Revised Cochrane Risk of Bias Tool for Randomized Trials (ROB2) was used for randomized studies. It assessed the quality of studies in 5 domains: randomization, deviations in the study process, missing data, outcome measures, and result reporting.

### Outcomes

The primary outcome was surgical site infection (SSI). Secondary outcomes included hospital length of stay (LOS), postoperative opioid consumption, pain scores, and postoperative nausea and vomiting (PONV).

## Results

### Included studies

A total of 150 records were identified through database searching. After removal of 31 duplicates, 119 studies underwent title and abstract screening, of which 78 were excluded. Forty-one full-text articles were assessed for eligibility. Of these, 4 studies met the final inclusion criteria after exclusion of non-AIS populations and noncomparative designs.

Fig. illustrates the PRISMA flow diagram of study selection.

**Fig. 1:**
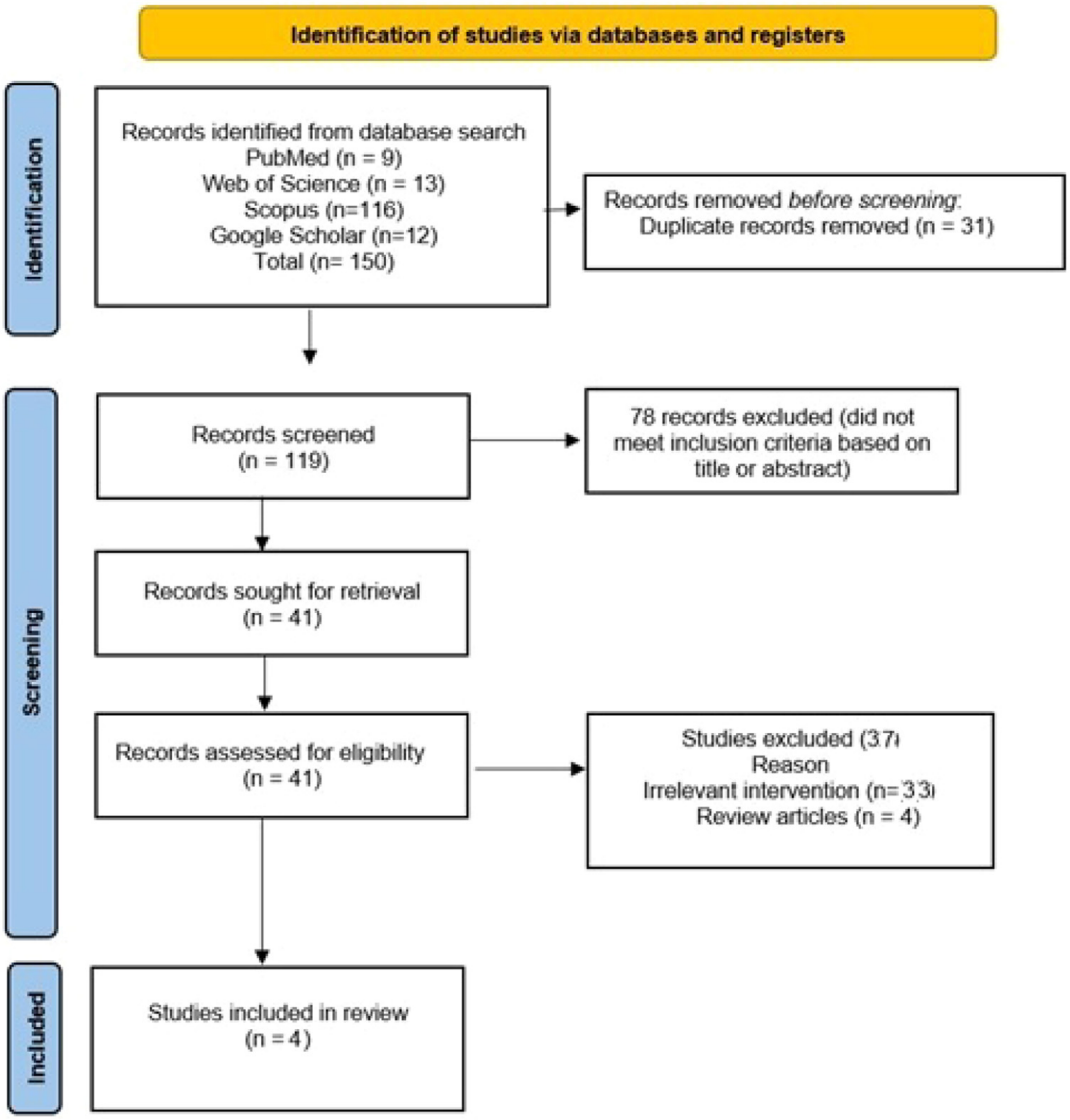
PRISMA flow diagram illustrating the study selection process.

### Study characteristics

The 4 included studies comprised a total of 614 patients with adolescent idiopathic scoliosis undergoing posterior spinal fusion. Study designs included retrospective and prospective comparative cohorts, as well as 1 randomized controlled trial (Table 1). All studies evaluated perioperative or postoperative dexamethasone administration compared with a control group.

**Table 1:** Summary of main findings of included studies.

**Author**	**Study design**	**Number of participants**	**Methodology**	**Dose**	**Infection rates**	**Other outcomes**
Mclntosh et al. [16]	Retrospective study	376	Participants were categorized into 3 cohorts: EPI=59, LB=149, and LB+D=168	IV 0.1–4 mg/kg per dose	SSI was observed in the LB cohort. No SSI was observed in the LB+D group.	Postoperative dexamethasone resulted in significantly lower LOS in hospital (p<.0001), opioid consumption, and pain scores in the LB+D group compared to the other groups (p<.0001).
Makino et al. [18]	Retrospective study	67	Patients were treated with PCA (n=35) Patients were given one-time sub-myofascial injections (a mixture of ropivacaine, epinephrine, and dexamethasone, n=32)	0.66 mg/kg	Nineteen patients from the control group and 14 from the sub-myofascial injection group required antiemesis.	The postsurgical pain score was lower in the group with sub-myofascial injection than in the control (p<.001). The WRT was also significantly lower in the injection group (p=.0007). Adverse effects and opioid use were reduced.
Fletcher et al. [19]	Prospective study	73	Patients randomized for the NS group (without postoperative steroids, n=65) or were managed with 3 doses (WS group, n=48)	8 mg per dose (median)	Three patients in the NS group and 4 patients in the WS group required additional wound dehiscence care (p=.53).	The use of postoperative steroids after PSF was linked with a 40% decrease in the use of postoperative opioids.
Wakamyia et al. [17]	Randomized controlled trial	98	All patients received anesthesia. After that, patients were given IV dexamethasone (n=48) or only 0.9% volume-equivalent saline (n=50).	0.15 mg/kg in 5 ml of 0.9% saline	Not reported	A single dose of dexamethasone reduced PONV in pediatric patients with adolescent idiopathic scoliosis (p=.02).

EPI, epidural; LB, liposomal plus plain bupivacaine; LB+D, liposomal, plain bupivacaine plus dexamethasone; PSF, posterior spinal fusion; PCA, patient-controlled anesthesia; PONV, postoperative nausea and vomiting; SSI, surgical site infection; LOS, length of stay.

Total participants: 614.

### Dexamethasone regimens

Dexamethasone dosing protocols varied substantially across studies. Reported regimens ranged from 0.1 to 4 mg/kg per dose administered intravenously to single-dose intravenous administration of 0.15 mg/kg. One study evaluated a sub myofascial injection containing dexamethasone at 0.66 mg/kg, while another administered 3 perioperative doses with a median of 8 mg per dose. The variability in dosing, timing, and route of administration precluded meaningful dose–response analysis (Table 2).

**Table 2:** Risk of bias measured with the Newcastle–Ottawa scale.

**Study**	**Selection**				**Comparability**		**Outcome**			**Total quality score**
	Representativeness of the exposed cohort	Selection of the nonexposed cohort	Ascertainment of exposure	Demonstration that the outcome of interest was not present at the start of the study	Controls for the most important risk factors	Controls for other risk factors	Assessment of outcome	Was follow-up long enough for outcomes to occur?	Adequacy of follow-up of cohorts	
Mclntosh et al. [16]	1	1	1	1	1	1	1	1	1	9
Makino et al. [18]	1	1	1	1	0	1	1	0	0	6
Fletcher et al. [19]	1	1	1	1	1	1	1	1	1	9

### Surgical site infection (primary outcome)

Across included AIS-specific studies, no statistically significant difference in surgical site infection rates was observed among patients receiving dexamethasone compared with controls. While isolated infection events were reported, differences between treatment and comparator groups did not reach statistical significance in individual studies. Given the limited sample sizes and low overall infection event rates, the included studies were likely underpowered to detect small differences in SSI risk.

### Postoperative recovery outcomes (secondary outcomes)

Dexamethasone administration was consistently associated with improved postoperative recovery parameters. Multiple studies reported reductions in opioid consumption and postoperative pain scores. Significant decreases in hospital length of stay were observed in comparative cohorts. Additionally, dexamethasone use was associated with lower rates of postoperative nausea and vomiting (PONV). However, heterogeneity in outcome definitions and reporting metrics limited quantitative pooling.

### Quality assessment

Risk-of-bias assessment demonstrated generally low to moderate methodological quality among the included AIS-specific studies. The randomized controlled trial showed low risk of bias across most domains, with minor concerns related to deviations from intended interventions.

Among observational studies, methodological quality ranged from moderate to high. The most common sources of bias were related to inadequate control of confounding variables and limited adjustment for baseline differences. In some studies, follow-up duration was insufficient to fully assess late complications.

Given the limited number of randomized trials and the predominance of retrospective designs, the overall strength of evidence remains moderate.

## Discussion

This systematic review evaluated the safety and efficacy of dexamethasone administration in adolescents with idiopathic scoliosis undergoing posterior spinal fusion. By restricting inclusion to AIS-specific populations and comparative study designs, this analysis reduces clinical heterogeneity and strengthens internal validity.

Across included studies, dexamethasone was consistently associated with reductions in postoperative opioid consumption, improved pain scores, decreased incidence of PONV, and shorter hospital length of stay. Importantly, no statistically significant increase in surgical site infection was identified. However, given the relatively small sample sizes and low event rates, the available studies may be underpowered to detect small differences in SSI risk.

Prior meta-analyses in broader surgical populations have demonstrated favorable analgesic and antiemetic effects of dexamethasone without clear increases in infection risk. While these findings provide contextual reassurance, AIS-specific evidence remains limited. The current review highlights a substantial gap in high-quality randomized trials evaluating perioperative steroid use in adolescent spinal deformity surgery.

A major limitation identified in the literature is the absence of standardized dosing protocols. Considerable variability in dose, timing, and route of administration precluded formal dose–response analysis. Therefore, the absence of statistical significance should not be interpreted as definitive evidence of safety. Future prospective trials should aim to define optimal dosing strategies while simultaneously evaluating potential effects on wound healing, fusion biology, and revision rates.

## Strengths and limitations

The principal strength of this review lies in its focused analysis of dexamethasone use specifically in adolescents undergoing posterior spinal fusion for idiopathic scoliosis. By limiting inclusion to AIS populations and comparative study designs, this review reduces clinical heterogeneity compared with prior iterations.

Previous studies and reviews have demonstrated that perioperative dexamethasone may reduce postoperative pain, opioid consumption, and postoperative nausea and vomiting across multiple surgical specialties [[8], [20], [21], [22], [23], [24], [25], [26], [27]].

Nevertheless, important limitations remain. The number of AIS-specific studies is limited, and most are retrospective in design. Significant variability in dexamethasone dosing protocols and outcome definitions prevented formal quantitative pooling and dose–response analysis. Furthermore, available studies were not uniformly powered to detect differences in rare outcomes such as surgical site infection.

## Conclusion

In adolescents with idiopathic scoliosis undergoing posterior spinal fusion, perioperative dexamethasone administration appears to improve postoperative recovery parameters, including pain control, opioid requirements, and PONV, However, current evidence remains inconclusive regarding its effect on surgical site infection risk due to limited sample sizes and low event rates. the limited number of AIS-specific studies, variability in dosing strategies, and predominance of nonrandomized designs underscore the need for adequately powered prospective trials. Standardization of dexamethasone dosing protocols and evaluation of long-term outcomes, including wound healing and fusion integrity, remain essential areas for future research.

## Author contributions

*Fahad H. Abduljabbar:* Formal analysis; Methodology; Project administration; Supervision; Validation; Visualization; Roles/Writing—original draft; Writing—review and editing; *Rakan Bokhari:* Formal analysis; Methodology; Project administration; Supervision; Validation; Visualization; Roles/Writing—original draft; Writing—review and editing; *Raed M. Sharaf:* Conceptualization; Data curation; Formal analysis; Investigation; Methodology; Project administration; Software; Supervision; Validation; Visualization; Roles/Writing—original draft; and Writing—review and editing; *Abdulrheem A. Almokhtar:* Formal analysis; Methodology; Project administration; Supervision; Validation; Visualization; Roles/Writing—original draft; Writing—review and editing; All authors reviewed and approved the final version of the manuscript.

## Data availability statement

The data supporting the findings of this case report are available from the corresponding author upon reasonable request

## Declaration of competing interest

The authors declare that they have no known competing financial interests or personal relationships that could have appeared to influence the work reported in this paper.

## Appendix Supplementary materials

Image, application 1
