# Supplementary material for: Postoperative dexamethasone administration following posterior spinal fusion for adolescent idiopathic scoliosis: A systematic review of worldwide data
Source: N Am Spine Soc J. 2026 Apr 18;26:100891. doi: 10.1016/j.xnsj.2026.100891 (PMC13224028; doi:10.1016/j.xnsj.2026.100891)
Supplement: Supplementary file 1 [file mmc1.docx]

**Supplementary Material**

**Keywords used in the database search**

| Key Variable | Subterms | Search options | PubMed | Web of Science | Scopus |
| --- | --- | --- | --- | --- | --- |
| 1. Dexamethasone | 1 Dexamethasone | All Fields | 85009 | 89991 | 456888 |
|  | 2 Dexamethasone | TI/AB | 69504 | 72288 | 77650 |
|  | 3 9α-Fluoro-16α-methylprednisolone | TI/AB | 4 | 1 | 4 |
|  | 4 Hexadecadrol | TI/AB | 38 | 6 | 42 |
|  | 5 Decadron | TI/AB | 153 | 136 | 207 |
|  | 6 DexPak | TI/AB | 2 | 0 | 2 |
|  | 7 Maxidex | TI/AB | 34 | 31 | 39 |
|  | 8 Ozurdex | TI/AB | 611 | 509 | 582 |
| (((((((Dexamethasone[Title/Abstract]) OR (9α-Fluoro-16α-methylprednisolone[Title/Abstract])) OR (Hexadecadrol[Title/Abstract])) OR (Decadron[Title/Abstract])) OR (DexPak[Title/Abstract])) OR (Maxidex[Title/Abstract])) OR (Ozurdex[Title/Abstract])) OR (Dexamethasone) | | | 85106 | 90165 | 456968 |
| 2. Scoliosis | 1 Scoliosis | Mesh | 22096 |  |  |
|  | 2 Scoliosis | TI/AB | 27867 | 23469 | 32703 |
|  | 3 posterior spine fusion | TI/AB | 197 | 5436 | 7437 |
|  | 4 lordosis | TI/AB | 10092 | 9095 | 11669 |
|  | 5 kyphosis | TI/AB | 12152 | 9876 | 13788 |
|  | 6 spinal curvatures | TI/AB | 353 | 2479 | 3696 |
| ((((("Scoliosis"[Mesh]) OR (Scoliosis[Title/Abstract])) OR (posterior spine fusion[Title/Abstract])) OR (lordosis[Title/Abstract])) OR (kyphosis[Title/Abstract])) OR (spinal curvatures[Title/Abstract]) | | | 46769 | 41362 | 56794 |
| 3. Posterior Spinal Fusion | 1 Posterior Spinal Fusion | All fields | 17353 | 12007 | 68904 |
|  | 2 Posterior Spinal Fusion | TI/AB | 2410 | 7319 | 9820 |
|  | 3 Posterior Lumbar Fusion | TI/AB | 539 | 5563 | 7427 |
|  | 4 Posterior Cervical Fusion | TI/AB | 398 | 3615 | 4874 |
|  | 5 Posterior Instrumented Spinal Fusion | TI/AB | 60 | 1198 | 1310 |
| (Posterior Spinal Fusion) OR ((((Posterior Spinal Fusion[Title/Abstract]) OR (Posterior Lumbar Fusion[Title/Abstract])) OR (Posterior Cervical Fusion[Title/Abstract])) OR (Posterior Instrumented Spinal Fusion[Title/Abstract])) | | | 17537 | 15456 | 69503 |
| (((Posterior Spinal Fusion) OR ((((Posterior Spinal Fusion[Title/Abstract]) OR (Posterior Lumbar Fusion[Title/Abstract])) OR (Posterior Cervical Fusion[Title/Abstract])) OR (Posterior Instrumented Spinal Fusion[Title/Abstract]))) AND ((((((((Dexamethasone[Title/Abstract]) OR (9α-Fluoro-16α-methylprednisolone[Title/Abstract])) OR (Hexadecadrol[Title/Abstract])) OR (Decadron[Title/Abstract])) OR (DexPak[Title/Abstract])) OR (Maxidex[Title/Abstract])) OR (Ozurdex[Title/Abstract])) OR (Dexamethasone))) AND (((((("Scoliosis"[Mesh]) OR (Scoliosis[Title/Abstract])) OR (posterior spine fusion[Title/Abstract])) OR (lordosis[Title/Abstract])) OR (kyphosis[Title/Abstract])) OR (spinal curvatures[Title/Abstract])) | | | 9 | 13 | 116 |
